# Amiodarone Therapy: Updated Practical Insights

**DOI:** 10.3390/jcm13206094

**Published:** 2024-10-12

**Authors:** Victorița Șorodoc, Lucia Indrei, Catinca Dobroghii, Andreea Asaftei, Alexandr Ceasovschih, Mihai Constantin, Cătălina Lionte, Bianca Codrina Morărașu, Alexandra-Diana Diaconu, Laurențiu Șorodoc

**Affiliations:** 1 Faculty of Medicine, Grigore T. Popa University of Medicine and Pharmacy, 700115 Iasi, Romania; 2 Second Internal Medicine Department, Sf. Spiridon Clinical Emergency Hospital, 700111 Iasi, Romania; 3 Radiology and Medical Imaging Department, Sf. Spiridon Clinical Emergency Hospital, 700111 Iasi, Romania

**Keywords:** amiodarone, atrial fibrillation, ventricular fibrillation, ventricular tachycardia, QT prolongation, amiodarone-induced pulmonary toxicity, amiodarone-induced hypothyroidism, amiodarone-induced thyrotoxicosis, blue-grey syndrome, amiodarone-induced keratopathy

## Abstract

Amiodarone, a bi-iodinated benzofuran derivative, is among the most commonly used antiarrhythmic drugs due to its high level of effectiveness. Though initially categorized as a class III agent, amiodarone exhibits antiarrhythmic properties across all four classes of antiarrhythmic drugs. Amiodarone is highly effective in maintaining sinus rhythm in patients with paroxysmal atrial fibrillation while also playing a crucial role in preventing a range of ventricular arrhythmias. Amiodarone has a complex pharmacokinetic profile, characterized by a large volume of distribution and a long half-life, which can range from several weeks to months, resulting in prolonged effects even after discontinuation. Side effects may include thyroid dysfunction, pulmonary fibrosis, and hepatic injury, necessitating regular follow-ups. Additionally, amiodarone interacts with several drugs, including anticoagulants, which must be managed to prevent adverse effects. Therefore, a deep understanding of both oral and intravenous formulations, as well as proper dosage adjustments, is essential. The aim of this paper is to provide a comprehensive and updated review on amiodarone’s indications, contraindications, recommended dosages, drug interactions, side effects, and monitoring protocols.

## 1. Introduction

Amiodarone, a potent antiarrhythmic medication with a complex pharmacological profile, has emerged as a pivotal therapeutic agent in the management of various cardiac arrhythmias, prompting extensive research into its mechanisms of action, clinical efficacy, and potential adverse effects [1]. Amiodarone is classically described as the prototype of class III antiarrhythmic drugs in the Vaughan Williams classification [2]. However, it also has class I, class II, and class IV properties, and none of these effects or their combination can fully explain its remarkable clinical effectiveness [1].

The objective of this article is to provide a detailed and comprehensive analysis of amiodarone, covering several key aspects of this medication. First, it will discuss its complex pharmacokinetics, pharmacodynamics and mechanism of action. Moreover, the article will address practical considerations regarding amiodarone dosing, emphasizing how doses should be adjusted based on clinical indications, in accordance with the latest guidelines. This approach ensures that the dosing recommendations are up-to-date and reflective of current best practices, enhancing the safety and efficacy of amiodarone therapy. Drug interactions will also be a key point of focus, given that amiodarone interacts with a wide range of medications, requiring careful attention from clinicians. In addition, the article will discuss newly emerging classes of drugs now being used in clinical practice, as well as updated data on potential interactions with amiodarone, ensuring a comprehensive understanding of how these developments may influence patient management and treatment outcomes. Both common and rare, but serious, adverse effects will be thoroughly discussed, with a particular emphasis on potential pulmonary, thyroid, cardiac, and liver complications. Finally, clear recommendations for patient monitoring during amiodarone therapy will be provided, stressing the importance of continuous supervision to minimize associated risks and ensure the treatment’s effectiveness.

For the purpose of this review, a comprehensive literature search was conducted using the PubMed and Embase databases. The search targeted articles offering in-depth insights into amiodarone. No restrictions were placed on publication years, allowing for the inclusion of both older and more recent studies to provide a thorough perspective on the topic. Particular emphasis was placed on identifying case reports, systematic reviews, and clinical trials that explored both acute and long-term risks associated with amiodarone use. Additionally, references from selected studies were examined to ensure the inclusion of all relevant and up-to-date research.

## 2. Pharmacokinetics, Pharmacodynamics, and Mechanism of Action

### 2.1. Pharmacokinetics

Amiodarone’s oral bioavailability is markedly inconsistent, ranging from 20% to 80% [3]. Furthermore, oral administration is characterized by extremely slow intestinal absorption, which can, however, be stimulated by a fat-rich diet. Co-administration of amiodarone with high-fat meals results in a notable enhancement of intestinal absorption, with an increase ranging from 2.4- to 3.8-fold compared to the fasting state [4].

Due to its substantial lipophilicity, the tissue distribution of amiodarone is influenced by distinct solubility profiles within various tissues, leading to a multi-compartmental distribution model encompassing central, peripheral, and deep compartments [5]. During the loading phase, a three-phase distribution pattern emerges: first, central or vascular distribution occurs within approximately 24 h (the central compartment is characterized by a low solubility coefficient and a limited volume of distribution); next, peripheral or solid organ distribution takes place over the following 7 days (the peripheral compartment—muscles, brain, thyroid—has an intermediate solubility coefficient and a relatively large volume of distribution); and finally, deep or adipose tissue distribution unfolds over the subsequent 4-week period (the deep compartment—adipose tissue, lungs, liver, and lymph nodes—has a high solubility coefficient and a markedly large volume of distribution). Thus, the deep compartment acts as an enormous reservoir for amiodarone storage [3,6]. The complete antiarrhythmic impact of amiodarone on ventricular arrhythmias reaches its plateau approximately 10 weeks into the course of therapy. The duration of medication persistence in the organism hinges on the fat composition and previous concentrations [7].

Amiodarone is eliminated from the body in a sequence opposite to its distribution, a process that spans several weeks, resulting in a half-life of 50 to 60 days [8]. Notably, the hepatic first-pass effect plays a pivotal role in amiodarone clearance, leading to the generation of mono-N-desethylamiodarone, a metabolite that retains antiarrhythmic properties similar to its parent compound [5]. Renal excretion plays a negligible role in the elimination process [3].

### 2.2. Pharmacodynamics

*Drug–channel interactions.* The relative blocking potency of amiodarone is high for repolarizing K^+^ channels, moderate for alpha and beta-adrenergic receptors (non-competitive blockade, with this effect being additive to the competitive receptor inhibition by beta-blockers), and low for Na^+^ channels (at high stimulation frequencies) and L-type (slow) Ca^+^ channels [9].

*Electrophysiological effects*. Amiodarone slows the heart rate, thereby prolonging the RR interval, lengthens the PR and QT intervals, while typically leaving the QRS duration unchanged. The drug prolongs the P-A, A-H, and H-V conduction times, as well as the atrial, atrioventricular (AV) nodal, and ventricular effective refractory periods. On the surface electrocardiogram (ECG), U waves are often observed, usually merging into the T waves [10].

*Non-electrophysiological properties (cardiac contractility, vascular tone, cardiac disease).* Amiodarone induces bradycardia and AV nodal inhibition, along with weak coronary and peripheral vasodilation. The weak negative inotropic effect is usually compensated for by the induced vasodilation [11].

### 2.3. Mechanism of Action

During the loading phase of acute intravenous (IV) administration, amiodarone exhibits more class I, IV, and non-selective class II effects, primarily blocking Na^+^ and Ca^+^ channels, as well as β-receptors, respectively [12]. The blockade of repolarizing K^+^ channels, which constitutes the class III effect, becomes evident after the completion of the loading dose, driven by elevated levels of the active metabolite, desethylamiodarone [13]. Thus, the oral formulation exerts a more pronounced class III effect following its conversion to desethylamiodarone [14].

Amiodarone slightly prolongs the depolarization phase (phase 0) of the action potential through a sodium channel blockade [13]. The elongation of the QT interval results from the lengthening of phases 2 and 3 of the action potential, achieved by blocking L-type calcium channels (LTCCs) and rectifier potassium channels (Kir), respectively [1].

While amiodarone does extend the QT interval, occurrences of torsade de pointes are infrequent, with an estimated incidence of merely 1–1.5% [6,15]. The safety and efficacy of amiodarone are likely bolstered by its complex electrophysiological effects. Notably, desethylamiodarone, which has comparable effects, might exhibit greater potency than amiodarone [16].

Another potential contributor to the antiarrhythmic effectiveness of amiodarone could be its effect on hindering the conversion of thyroxine (T4) into triiodothyronine (T3) [17]. Anticipated outcomes in thyroid function tests include normal or slightly elevated levels of thyroid-stimulating hormone (TSH), reduced T3 levels, and elevated levels of both T4 and reverse T3. These alterations generally occur without significant clinical consequences [18].

## 3. Indications and Dosing

Amiodarone is widely recognized for its numerous therapeutic indications, being employed in both acute and chronic clinical contexts. Its versatility allows it to be considered either as a first-line or second-line treatment option, depending on the specific condition being managed. In many cases, other antiarrhythmic drugs (AADs) may also be suitable alternatives, necessitating a thoughtful selection between amiodarone and other available therapies.

The various clinical contexts in which amiodarone is indicated, whether as a first-line or second-line therapy, along with other recommended medications for those specific situations, are outlined in Table 1.

### 3.1. Acute Use in Emergency Medicine and Critical Care

#### 3.1.1. Cardiac Arrest, Ventricular Fibrillation (VF), Hemodynamically Unstable Ventricular Tachycardia (VT)

The recommended dose of amiodarone in this setting is an initial IV dose of 300 mg, with a second IV dose of 150 mg if required [22].

In out-of-hospital cardiac arrest caused by VF, parenteral amiodarone may improve survival to admission but does not seem to significantly impact survival or neurological outcomes at discharge [23,24].

In a study comparing amiodarone, lidocaine, and placebo, active medications (amiodarone or lidocaine) improved survival rates in bystander-witnessed cardiac arrests compared to a placebo [25]. This benefit was not observed in unwitnessed arrests, likely due to faster drug administration in witnessed cases. There was no significant difference in survival between amiodarone and lidocaine. However, more patients on amiodarone required temporary cardiac pacing. Neither amiodarone nor lidocaine showed a significant increase in survival or neurological outcomes over a placebo in shock-refractory VF or pulseless VT [25].

Consequently, the 2018 American Heart Association (AHA) Focused Update on Advanced Cardiovascular Life Support recommends considering the administration of amiodarone or lidocaine to patients with VF/pulseless VT unresponsive to defibrillation, particularly in the setting of a witnessed cardiac arrest, when the interval from the event to drug administration is likely shorter [19]. Moreover, IV amiodarone appears to play a role in reducing the occurrence of recurrent VT or VF during resuscitation [19].

Despite the limited data, the combined use of beta-blockers and amiodarone emerges as the most effective pharmacological treatment strategy for managing electrical storm [6,26]. The European Society of Cardiology (ESC) endorses this antiarrhythmic therapy, recommending a non-selective beta-blocker alongside IV amiodarone in patients presenting with electrical storm and structural heart disease, in the absence of contraindications (e.g., severely reduced left ventricular ejection fraction or blood pressure may preclude the use of beta-blockers) [20].

#### 3.1.2. Stable VT

The recommended dose of amiodarone in this context is a 150 mg bolus, followed by an infusion of 1 mg/min for 6 h, then 0.5 mg/min for 18 h [20].

To pharmacologically terminate a hemodynamically tolerated VT, the ESC recommends either IV amiodarone or procainamide, while the American College of Cardiology (ACC) considers the administration of intravenous amiodarone or sotalol [20,22].

The PROCAMIO study randomized patients with wide QRS tachycardia to receive either procainamide (10 mg/kg over 20 min) or amiodarone (5 mg/kg over 20 min). The results showed that procainamide had fewer major cardiac adverse events, was more effective in terminating tachycardia, and was preferred over amiodarone, especially in patients with structural heart disease [27].

#### 3.1.3. Atrial Fibrillation (AF)—Acute Cardioversion

The starting dose for cardioversion is 300 mg administered IV over 30–60 min, followed by an additional dose of 900–1200 mg IV over 24 h, or alternatively, 200 mg orally three times a day for 4 weeks. The acute success rate is 44% (higher in recent-onset AF), with the expected time to sinus rhythm ranging from 8–12 h to several days [21]. Pharmacological cardioversion of AF is indicated only in hemodynamically stable patients, considering the thromboembolic risk. While less effective than electrical cardioversion, the pharmacological approach does not require patient sedation. The ESC recommends IV amiodarone for cardioversion in patients with AF associated with structural heart disease or heart failure, provided that delayed cardioversion aligns with the patient’s clinical context due to the drug’s delayed effect [21]. Additionally, evidence suggests that administering amiodarone prior to elective electrical cardioversion can enhance its effectiveness [28].

When performing cardioversion, due to the increased thromboembolic risk, patients must be anticoagulated. If the patient has already been on anticoagulation for at least 3 weeks, cardioversion can proceed either pharmacologically or electrically. If the patient has not been on prior anticoagulation and the situation allows, anticoagulation should be initiated for at least 3 weeks before cardioversion [21]. In cases where urgent cardioversion is required, anticoagulation should be initiated promptly, and cardioversion can be performed depending on the timing of AF onset. If the AF onset is clearly within the last 24 h, cardioversion can proceed immediately. However, if the onset is uncertain or has been longer than 24 h, additional precautions such as a transesophageal echocardiogram (TOE) are necessary to assess for the presence of thrombi before performing cardioversion [21].

#### 3.1.4. AF—Acute Rate Control

In cases of hemodynamic instability or significantly reduced left ventricular ejection fraction, IV amiodarone may be considered for acute heart rate control [29]. The suggested administration involves 300 mg of IV amiodarone, diluted in 250 mL of 5% dextrose and infused over 30–60 min, ideally through a central venous line. This is followed by a dose of 900–1200 mg IV over 24 h, diluted in 500–1000 mL, through a central venous line [21].

When using amiodarone for acute rate control in patients with AF, if they have not already been anticoagulated, anticoagulation should be initiated due to the associated thromboembolic risk, which is especially elevated if conversion to sinus rhythm occurs [21].

#### 3.1.5. Other Supraventricular Tachyarrhythmias

In situations where vagal maneuvers and adenosine are ineffective for managing wide QRS tachycardia without a confirmed diagnosis, IV amiodarone may be considered, provided the patient’s hemodynamic stability is maintained [30].

Amiodarone may be used as acute therapy (IV) in hemodynamically stable patients and as a chronic therapy (orally) for the treatment of focal atrial tachycardia and macro-re-entrant atrial arrhythmias, but only as a last resort [30].

IV amiodarone may also be considered in refractory cases of antidromic AV re-entrant tachycardia [29].

Amiodarone is potentially harmful and is therefore contraindicated in pre-excited AF [21,30].

### 3.2. Chronic Use for Long-Term Management

#### 3.2.1. Prevention of Recurrent Implantable Cardioverter-Defibrillator (ICD) Shocks and Ventricular Tachycardia

Among individuals with ischemic cardiomyopathy and an ICD who experienced VT despite antiarrhythmic drug treatment, those who underwent catheter ablation had a significantly reduced frequency of the combined primary endpoint (death, VT storm, or appropriate ICD shock) compared to those with escalated drug therapy [31]. This benefit was notable only in patients whose arrhythmia persisted despite baseline amiodarone therapy. No significant difference in outcomes was observed between groups in patients on non-amiodarone antiarrhythmic drugs [31].

#### 3.2.2. AF—Prevention of Recurrent AF

As a long-term AAD therapy for rhythm control, the oral dose is 3 × 200 mg daily for 4 weeks, then 200 mg daily [21]. Since amiodarone is the most effective antiarrhythmic drug for maintaining sinus rhythm, with fewer recurrences of AF compared to dronedarone or sotalol, the ESC recommends its use for long-term rhythm control in all patients with AF, including those with heart failure and reduced ejection fraction [21]. However, due to its potential for extracardiac toxicity, alternative AADs should be prioritized whenever feasible [32,33,34].

#### 3.2.3. AF—Rate Control

The usual oral maintenance dose is 200 mg once daily after loading with 3 × 200 mg daily for 4 weeks, then 200 mg daily (adjust other rate-controlling drugs according to heart rate). Amiodarone serves as a final option for heart rate management when combination therapy fails in patients ineligible for non-pharmacological rate control methods, such as AV node ablation and pacing, despite its extracardiac side effects [21].

## 4. Drug Interactions

### 4.1. Cardiovascular Medication

*Class Ia antiarrhythmic drugs (amiodarone increases the serum concentration of both procainamide and quinidine) and other class III antiarrhythmics*. Increased risk of QT prolongation and torsade de pointes. Amiodarone should not be administered in conjunction with these drugs [35].

*Flecainide.* Flecainide is metabolized by cytochrome P450 2D6 (CYP2D6), and concomitant administration of amiodarone increases serum flecainide levels. Although flecainide and amiodarone are rarely used together in clinical practice, it is recommended to reduce the flecainide dose by 50% when the patient is also receiving amiodarone [35,36].

*Beta-blockers and non-dihydropyridine calcium channel blockers.* Concomitant use with amiodarone may cause sinus bradycardia or AV block. The presence of bradycardia is notably linked to the use of amiodarone and any medication aimed at controlling heart rate. Among individuals on amiodarone, bradycardia is most strongly correlated with the use of rate-controlling medications and being female [37]. Consequently, caution is advised when prescribing dual therapy with amiodarone and beta-blockers or amiodarone and non-dihydropyridine calcium channel blockers [35].

*Digoxin.* Digoxin bypasses cytochrome P450 (CYP450) metabolism, with alterations in its absorption and elimination primarily influenced by P-glycoprotein (P-gp) efflux function in the intestines and kidneys. Given digoxin’s narrow therapeutic range, vigilant observation for signs of digitalis-related toxicity is imperative [38]. Several co-medications, notably established P-gp inhibitors like quinidine, amiodarone, and verapamil, have been documented to elevate digoxin concentrations beyond its safe upper limit. Therefore, it is recommended to reduce the digoxin dose by 50% while closely monitoring its serum concentration levels [39].

*Statins (Lovastatin, Simvastatin).* Co-administration with amiodarone may increase the risk of rhabdomyolysis or myopathy. The Study of the Effectiveness of Additional Reductions in Cholesterol and Homocysteine (SEARCH) demonstrated a relatively high incidence of myopathy (approximately 6%) in patients using simvastatin and amiodarone concurrently, although the precise cause of this interaction remains unclear [40]. Numerous case reports have linked rhabdomyolysis to the use of both simvastatin and amiodarone, indicating that amiodarone may enhance the risk of rhabdomyolysis through CYP3A4 inhibition, leading to reduced simvastatin clearance and elevated plasma levels, potentially reaching toxic levels [41]. The study by Becquemont et al. highlights amiodarone’s interaction with simvastatin, but not pravastatin. Consequently, when amiodarone and a 3-hydroxy-3-methylglutaryl-Coenzyme A reductase inhibitor are co-prescribed, pravastatin should be preferred over simvastatin to prevent potential drug interactions [42].

*Warfarin.* While most patients maintain therapeutic warfarin doses, over a third of those starting concurrent amiodarone treatment experienced excessive anticoagulation within three weeks. To minimize the risk of severe and avoidable bleeding, the initiation of amiodarone should be accompanied by intensified INR monitoring, along with an expected average dose reduction of warfarin by 25–33% [43].

*Direct-oral anticoagulants (DOACs).* Given the frequent association between amiodarone and DOACs in the context of AF, it is crucial to understand the interactions between these two drug classes. The absorption, metabolism, and elimination of DOACs rely on the P-gp transporter system and CYP3A4 enzymes. Amiodarone, as a moderate inhibitor of both P-gp and CYP3A4, can lead to increased serum levels of DOACs (varying by specific drug), potentially increasing the risk of bleeding [44]. The available data on the bleeding risk in AF patients receiving both amiodarone and DOACs remains insufficient and is sometimes even contradictory [44,45,46]. Due to concerns about drug–drug interactions, the 2021 European Heart Rhythm Association Practical Guide on the Use of Non-Vitamin K Antagonist Oral Anticoagulants in Patients with Atrial Fibrillation introduced a color-coded interaction table for DOACs, which also includes clinical risk factors that may increase bleeding risk [47]. The guide advises caution with DOAC dosing when at least two yellow risk factors are present, recommending the use of alternative medications with fewer interactions or selecting a DOAC that has less potential for interactions. If these options are not feasible, alternative stroke prevention strategies or off-label DOAC dose reductions may be considered [47].

*Sodium glucose co-transporter-2 (SGLT2) inhibitors.* Initially meant as antidiabetic drugs, SGLT2 inhibitors are currently one of the most important pillars in the treatment of chronic heart failure (HF), these drugs being known to reduce cardiovascular outcomes, including mortality, also lowering the risk of HF-related hospitalization [48]. Studies have also shown that they, especially empagliglozin and dapagliflozin, may have benefits in HF patients by reducing arrhythmias. SGLT2 inhibitors demonstrate antiarrhythmic effects through several key mechanisms. They reduce the late sodium current, preventing action potential prolongation and stabilizing myocardial electrical activity [49]. Additionally, these drugs have been found to modulate ion channels, particularly potassium and calcium, which helps restore normal ion channel function and reduces the risk of arrhythmias [50]. SGLT2 inhibitors also protect mitochondrial function and lower inflammation, preventing structural and electrical remodeling of the heart that can lead to arrhythmias [51]. Furthermore, they reduce epicardial fat and its associated adipokine secretion, which otherwise contributes to arrhythmogenic changes [52]. Large clinical trials and meta-analyses have revealed that SGLT2 inhibitors can reduce the incidence of atrial and ventricular arrhythmias in patients with HF, regardless of whether they have diabetes. These findings highlight the broad cardiovascular benefits of SGLT2 inhibitors beyond glucose control, with ongoing trials expected to provide more evidence on their antiarrhythmic potential [50]. For example, one study demonstrated that empagliflozin reduces the recurrence of AF in diabetic patients with paroxysmal or persistent AF. This was shown through a case-crossover analysis where patients’ AF episodes were compared one year before and after starting empagliflozin treatment. The results revealed a significant decrease in AF recurrence after treatment. The authors suggest that while the exact mechanisms of empagliflozin’s antiarrhythmic effects remain unknown, they believe it may be linked to the drug’s influence on several AF risk factors, including myocardial remodeling, blood pressure regulation, body weight reduction, inflammation, oxidative stress, and decreased adrenergic activity [53]. The simultaneous use of amiodarone and SGLT2 inhibitors may raise concerns due to their different effects on cardiac electrophysiology. While combining these drugs could enhance antiarrhythmic effects, there is a need for careful monitoring, as both can influence myocardial ion balance. In particular, clinicians should be cautious about potential disturbances in electrolyte levels, especially potassium, which could increase the risk of arrhythmias. Although there are limited data on specific interactions between these drugs, further research is needed, and close patient monitoring is recommended when they are prescribed together.

### 4.2. Non-Cardiovascular Medication

*Macrolide antibiotics, fluoroquinolones, azole antifungals, fluoxetine.* These drugs are known to predispose individuals to QT prolongation. Combining amiodarone with such medications can lead to an R-on-T phenomenon and increase the risk of torsade de pointes. It is recommended to avoid these combinations (risk category X) [1].

*Cholestyramine.* Amiodarone metabolism is characterized by biliary excretion and enterohepatic circulation. Cholestyramine significantly reduces the enterohepatic circulation of both amiodarone and its metabolite desethylamiodarone, leading to an increased (by 10–20%) and more rapid elimination of the antiarrhythmic drug [54]. This aspect can be particularly useful when faced with significant side effects from amiodarone administration, as high doses of cholestyramine might help mitigate these side effects [55].

*Cyclosporine.* Shared metabolic pathways between cyclosporine and amiodarone suggest a plausible drug interaction. To prevent possible drug-related toxicity, reducing the cyclosporine dosage might be essential during co-administration with amiodarone. Close monitoring of cyclosporine levels is advised after amiodarone dose reduction, with potential cyclosporine adjustments weeks later due to amiodarone’s prolonged half-life [56,57].

*Antiretrovirals.* The co-administration of amiodarone with antiretroviral (ARV) therapy is complex due to shared involvement in the CYP450 enzyme system. ARVs can act as substrates, inhibitors, or inducers, affecting glucuronidation and P-gp transport [58]. Amiodarone’s inhibition of CYP3A4 and P-gp may lead to ARV accumulation [59]. Significant interactions have been observed with protease inhibitors (PIs) and non-nucleoside reverse transcriptase inhibitors (NNRTIs), influencing serum concentrations. NNRTIs like efavirenz and rilpivirine may cause QT prolongation and cardiotoxicity. ARVs that increase amiodarone serum levels include atazanavir, darunavir, fosamprenavir, indinavir, lopinavir, nelfinavir, saquinavir, and elvitegravir. Nucleoside reverse transcriptase inhibitors (NRTIs), raltegravir, and dolutegravir are generally safe with amiodarone [59]. Using an ARV regimen with two NRTIs plus raltegravir, dolutegravir, or maraviroc can limit amiodarone’s impact. If co-administration is necessary, regular monitoring every 4–6 weeks, along with frequent ECG monitoring, is recommended [59].

*Rifampicin.* This drug is a potent inducer of the P450 enzyme system and has been reported to lead to subtherapeutic amiodarone serum concentrations [60]. The onset of CYP3A4 induction by rifampicin experiences a delay and reaches its maximal impact around 15 days. Given the prolonged and variable elimination half-lives of approximately 50 days for amiodarone and 60 days for desethylamiodarone, predicting the outcomes of this drug–drug interaction in individual patients is complex [61]. In summary, rifampicin effectively induces amiodarone metabolism, causing subtherapeutic concentrations within 1–2 weeks and necessitating dose adjustments. Therapeutic drug monitoring proves beneficial for managing this interaction [61].

*Fentanyl.* This drug is also a CYP3A substrate. Co-administration of amiodarone and fentanyl may lead to bradycardia, decreased cardiac output, and hypotension [36].

*Glucagon-like peptide 1 (GLP-1) receptor agonists.* GLP-1 receptor agonists are primarily indicated for the treatment of type 2 diabetes to improve glycemic control by stimulating insulin secretion and inhibiting glucagon release. They are also indicated for weight management in patients with obesity or overweight, particularly those with type 2 diabetes [62]. Additionally, GLP-1 receptor agonists have shown cardiovascular benefits and are recommended for reducing the risk of major cardiovascular events in patients with type 2 diabetes who have established cardiovascular disease or are at high cardiovascular risk [63]. Available data suggest that GLP-1 receptor agonists show potential antiarrhythmic effects in AF by addressing its underlying metabolic and inflammatory causes [64]. These drugs help stabilize blood glucose levels, reduce epicardial fat, and control weight, all of which are linked to AF risk. They also reduce oxidative stress and inflammation, key contributors to atrial remodeling, and regulate calcium metabolism, which plays a critical role in cardiac electrical activity. Additionally, GLP-1 RAs improve mitochondrial function and energy efficiency in heart cells, which can help prevent atrial electrical and structural changes that promote AF [64,65]. Karakasis et al. conducted a meta-analysis to evaluate the effects of GLP-1 receptor agonists on the recurrence of AF after catheter ablation. The analysis included data from three studies involving 6031 participants. The findings revealed that GLP-1 receptor agonists significantly reduced AF recurrence post-ablation, with a 45% lower risk compared to control groups. Although further research is needed, these findings suggest that GLP-1 receptor agonists could play a significant role in managing AF, especially in post-ablation patients [66]. Although direct drug interactions between GLP-1 receptor agonists and amiodarone are not well-documented, the combined impact on cardiovascular health and electrolyte balance warrants careful monitoring, especially in patients with a high risk of arrhythmias. Further research may provide more insights into the safety and efficacy of using these treatments together. Close monitoring is also necessary in cases of significant weight loss induced by GLP-1 receptor agonists, as this can affect drug distribution. This recommendation is based on an observation by Bourron et al. who reported a case of amiodarone-induced thyrotoxicosis in a patient who experienced a 45% weight loss following a Roux-en-Y gastric bypass. Such findings highlight the potential for altered drug effects after substantial weight reduction, necessitating careful supervision [67,68].

## 5. Side Effects and Their Management

### 5.1. Cardiovascular System

Amiodarone is contraindicated in cases of severe sinus node dysfunction with notable sinus bradycardia or syncope, second- or third-degree heart block, known hypersensitivity, cardiogenic shock, and likely severe chronic lung disease. The prevalent adverse effects encompass sinus bradycardia, particularly in older individuals, and QT prolongation [2].

However, the occurrence of torsades de points is extremely rare [2]. This can be explained by amiodarone’s unique mechanism of action. Amiodarone has properties of all four classes of antiarrhythmics and is unique from other class III drugs. It blocks multiple ion channels, including potassium, sodium, and calcium, which prolong the action potential without significantly increasing the dispersion of ventricular repolarization [1,2]. This ability to stabilize and promote uniform repolarization across the myocardial layers reduces the risk of chaotic electrical activity that could lead to torsades de points. Additionally, the reduced likelihood of amiodarone causing torsades de points may also be linked to its ability to inhibit early after-depolarizations, events that are known to contribute to prolonged repolarization and increase the risk of ventricular arrhythmias [15].

Cardiac monitoring for QT prolongation and bradycardia is advised prior to initiating amiodarone and annually thereafter [16]. In cases of asymptomatic bradycardia, maintaining the dose is recommended if the heart rate exceeds 40 beats per minute [1]. However, if heart rates fall below 40 beats per minute, a 50% dose reduction is suggested, with subsequent monitoring for breakthrough tachyarrhythmias and improved sinus heart rate using a 14- or 30-day event monitor. In the event of symptomatic bradycardia, amiodarone dosage should be reduced by 50% or its administration should be temporarily suspended. If intolerable tachyarrhythmias arise, considering pacemaker placement for supraventricular tachycardias or an ICD for VT patients is appropriate [1].

The initial approach to amiodarone-triggered polymorphic VT involves the swift cessation of amiodarone and addressing concurrent factors that facilitate ventricular arrhythmia, notably correcting electrolyte imbalances and bradycardia [69]. It is crucial to recognize that VT can emerge early or late during treatment with a medication prone to causing this arrhythmia. This could result from toxic drug levels or the emergence of “triggering” elements, such as electrolyte disturbances, even in the presence of normal or slightly elevated drug levels [70].

While evaluating the QT interval has become a routine practice during in-hospital monitoring, there is a lack of consensus regarding specific protocols, including measurement techniques (manual, semi-automated, fully automated continuous), heart rate correction methods, measurement frequency, and patient inclusion criteria [71]. Due to limited conclusive research, a significant portion of the provided recommendations is based on expert viewpoints. Assessing QT in patients with AF poses particular challenges during manual measurement due to the continuously changing RR interval [72]. Although no consensus exists on the optimal approach, multiple methods have been proposed. One method involves averaging the longest and shortest QT intervals in an ECG recording. Another method calculates the average of multiple QT measurements (up to 10) in a recording [73]. Approaches relying on a single QT measurement are less likely to accurately represent repolarization duration [72].

The AHA/ACC Foundation/Heart Rhythm Society guidelines on ECG standardization and interpretation suggest selecting the ECG lead with the lengthiest T wave for QT interval monitoring, avoiding leads with U waves. Consistency is key, using the same lead for a patient over time, as QT duration varies across the 12 leads [74]. The QT interval is measured from the QRS complex onset to the T wave termination. In instances of prolonged QRS, such as with new bundle branch block (BBB), the resultant QT increase should not be taken as acquired long QT syndrome (LQTS). If T waves are notched, biphasic, or if U waves are superimposed, the end of the entire T-wave complex should define the QT interval’s termination. For distinct U waves occurring after the T wave’s return to baseline, they should not be included in the QT measurement [74].

As the QT interval elongates with slow heart rates and shortens with rapid heart rates, it is crucial to adjust the QT interval for heart rate to precisely identify repolarization changes over time [75]. The Bazett formula, where the measured QT interval is divided by the square root of the R-R interval (in seconds), is the widely employed QT correction method. Yet, several studies reveal that the Bazett approach overestimates corrected QT (QTc) values during higher heart rates [73]. Various alternatives, like the Hodges, Framingham, Fridericia, and subject-specific formulas, have demonstrated greater accuracy. While consensus on the ultimate formula is currently lacking, some of these alternatives may gain prominence in the future [76]. The QTc interval is deemed prolonged when it exceeds 450 milliseconds in male patients and 460 milliseconds in female patients. This sex difference diminishes around 40 years of age [77].

An examination of research and expert perspectives underscores that a QTc duration exceeding 500 milliseconds is linked to an elevated risk of torsades de pointes. While criteria for QTc prolongation have been suggested, a definitive threshold indicating QTc prolongation without proarrhythmic risk remains uncertain [78,79].

It is important to clarify that a mild QT interval prolongation, such as below 500 ms, in asymptomatic patients is not necessarily dangerous and is, in fact, related to the intended pharmacologic effect of amiodarone. The drug works by prolonging the action potential duration, which is crucial for controlling certain types of arrhythmias [1,9]. This prolongation should not be interpreted as an adverse side effect but rather as a manifestation of the drug’s mechanism of action. Generally, the risk of torsades becomes significant only with extreme QT prolongation or in the presence of other predisposing factors, such as electrolyte imbalances or the concomitant use of other medications that affect repolarization [80,81].

### 5.2. Lungs

Amiodarone-induced pulmonary toxicity (APT) primarily manifests as interstitial lung disease or hypersensitivity syndrome, with a relative risk of 1.77 compared to a placebo. However, cases of acute respiratory distress syndrome, pulmonary nodules/masses, and pleural effusions have also been documented [82,83,84,85,86].

APT should not be confused with amiodarone’s effect on the lungs, which is usually asymptomatic and known as lipoid pneumonia. The occurrence rate of APT ranges from approximately 4% to 17% among patients, with a fatality rate of about 10% [87]. This rate is influenced by factors such as dosage, patient age, and pre-existing lung conditions [88]. The incidence levels off when the cumulative dose surpasses 150 g [89]. Studies focusing on an amiodarone dose of 200 mg per day showed no statistically significant difference in the occurrence of pulmonary adverse events between the amiodarone and placebo groups, underlining the importance of the dose-related effect [82].

Additionally, APT onset appears to be facilitated by underlying pathologies, general anesthesia, oxygen administration, and invasive or surgical procedures. It is important to note that acute pulmonary side effects can also occur even after short-term administration. While these are less common, cases of ARDS, eosinophilic pneumonia, and diffuse alveolar damage have been reported, particularly in patients with pre-existing pulmonary conditions or those undergoing procedures that involve oxygen therapy [90,91].

For example, a case was reported where a 61-year-old patient with chronic obstructive pulmonary disease (COPD) and severe heart failure developed ARDS shortly after being started on amiodarone for AF. Despite initial stabilization, his condition worsened after the introduction of amiodarone, as evidenced by the progression of ground-glass opacities on CT scans. The patient was successfully treated with corticosteroids after a diagnosis of APT. This case highlights the possibility of acute onset pulmonary toxicity, even in the absence of prolonged exposure to amiodarone [92].

Another example for how pulmonary complications can arise quickly after amiodarone initiation, especially in patients with underlying lung conditions, is that of a 72-year-old man with COPD and heart failure. After three weeks of amiodarone treatment, he developed ARDS. Despite discontinuing the drug and receiving corticosteroids, he ultimately died from respiratory failure [93].

While cases of acute pulmonary toxicity after a single dose of amiodarone are not reported in the literature, there are instances of significant pulmonary side effects arising shortly after amiodarone initiation, particularly in patients with pre-existing lung conditions like COPD. These cases highlight the need for close monitoring and early intervention in vulnerable patients to prevent rapid deterioration. Given the potential risks, it is crucial to remain vigilant when starting amiodarone, especially in individuals with underlying respiratory issues.

The mechanism through which amiodarone induces lung toxicity is thought to involve stimulation of the angiotensin enzyme system, leading to the apoptosis of pneumocytes [94,95]. The drug’s toxic effects are believed to result from its active metabolite combining with lipids and accumulating in lung macrophages, leading to direct inflammation and damage to lung cells [94]. This affects structural cells, specifically type II pneumocytes, causing cytotoxic effects. Immune-mediated mechanisms are indicated by T-cell accumulation, which also triggers apoptosis [96]. Bronchoalveolar lavage may reveal alveolar septae widening, interstitial fibrosis, cytotoxic T cells, and type II pneumocyte hyperplasia. Microscopic lung tissue examination might show “laminated” inclusion bodies [97]. Essentially, amiodarone appears to both structurally alter the lung and cause cellular destruction. A study conducted by Kosseifi et al. suggests that concomitant treatment with an angiotensin-converting enzyme (ACE) inhibitor or an angiotensin receptor blocker (ARB) reduces the risk of developing APT in patients treated with amiodarone [98].

A definitive diagnostic test specific to APT is lacking. Rather, diagnosis relies on excluding other possibilities through clinical, laboratory, and radiographic assessments. All individuals on amiodarone treatment should undergo chest X-ray and liver function tests before initiation, followed by vigilant monitoring [94]. APT can present diversely, ranging from subacute alveolar/interstitial pneumonitis to severe respiratory distress with profound hypoxemia [99]. Laboratory findings might indicate non-specific increases in white blood cell (WBC) count, erythrocyte sedimentation rate (ESR), and lactate dehydrogenase (LDH). Some authors propose evaluating KL-6 serum levels, a glycoprotein often elevated in interstitial pneumonitis cases, though its utility as a specific marker for APT remains uncertain [100,101]. In a clinically suggestive context, diagnosis heavily relies on chest X-rays and high-resolution computed tomography (HRCT), revealing changes such as ground-glass opacities, areas of fibrosis, or nodules in the lung parenchyma [94]. Lung function tests may show a restrictive syndrome along with impaired diffusing capacity of the lungs for carbon monoxide (DLCO) [100].

Swift diagnosis enables patients to respond positively to discontinuation of amiodarone and the administration of steroids. It is generally agreed that corticosteroid therapy is advisable for patients exhibiting extensive damage (as observed in radiology reports) or hypoxemia to prevent lung fibrosis [87,102]. Amiodarone toxicity typically requires high-dose, extended corticosteroid treatment. However, due to the limited availability of controlled trials, recommendations regarding dosage and duration are constrained. Numerous studies suggest prednisone at a dose of 0.5 to 1 mg/kg for up to 12 months [103]. When discontinuing amiodarone, some experts recommend a slower corticosteroid taper for patients with an elevated body mass index (BMI) to mitigate the risk of relapse, given the drug’s pronounced lipophilic nature. The elevated drug concentration in adipose tissues causes patients with higher BMIs to have elevated serum amiodarone levels for a significantly extended period compared to those with normal BMIs [99].

### 5.3. Liver

Long-term administration of amiodarone is associated with two different types of liver toxicity. Asymptomatic elevation of transaminases is frequent and usually transient. Amiodarone-induced hepatitis, cirrhosis, and liver failure are far less common [104]. Histologically, the hepatitis is similar to alcoholic hepatitis (steatosis, fibrosis, phospholipid-laden lysosomal lamellar bodies). Medical practitioners should perform initial and subsequent assessments of liver function every six months due to the possibility of hepatic impairment [105]. These evaluations should encompass aspartate aminotransferase (AST) and alanine aminotransferase (ALT) levels. In cases where AST or ALT elevation exceeds twice the upper normal limit, adjustments like amiodarone dose reduction or transitioning to a different agent should be considered [6,106].

Occasionally, albeit with a potentially fatal outcome, there is an infrequent manifestation of symptomatic liver injury linked to the IV administration of amiodarone [107]. Available records suggest that hepatotoxicity tends to manifest within 24 h of initiating IV amiodarone, although in certain cases, it may appear after more than three days [108]. Typically, liver function gradually improves upon discontinuing IV amiodarone. This form of toxicity predominantly involves hepatocellular injury rather than cholestasis [109]. The precise mechanism of hepatotoxicity stemming from IV amiodarone remains uncertain. Some studies propose mitochondrial damage as a contributing factor to amiodarone’s toxicity [110]. Notably, resuming oral amiodarone did not exacerbate hepatic injury in certain instances [111], leading Rhodes et al. to hypothesize that the origin of hepatotoxicity might not be the amiodarone itself, but rather other constituents present in the amiodarone infusion [112].

Amiodarone, characterized by its lipophilic nature, is formulated as an IV solution using a combination of polysorbate 80 and a minor quantity of benzyl alcohol to achieve stability [113]. It is worth noting that polysorbate 80 has been linked to the E-ferol syndrome, a condition with clinical manifestations akin to those observed in cases of liver toxicity associated with IV amiodarone [114]. This intriguing similarity raises the possibility that the hepatic injury could potentially stem from the presence of polysorbate rather than the amiodarone compound itself [112].

The dosage administered could emerge as a significant factor contributing to the development of hyperacute hepatitis. Notably, instances of uneventful resumption of oral amiodarone have been marked by substantially lower dosages compared to the IV form [111]. Amiodarone’s primary metabolic pathways involve CYP450 enzymes (CYP3A4 and CYP2C8). N-desethylamiodarone has been implicated as a potential instigator of hepatotoxicity [110]. Additionally, the propensity for amiodarone-related mitochondrial toxicity is also contingent upon the dosage administered. Consequently, the likelihood of inducing hyperacute hepatitis remains plausible with high oral amiodarone dosages, despite this phenomenon appearing to be more exclusive to the IV form [115].

For patients experiencing hepatic injury, prompt discontinuation of IV amiodarone is recommended, with avoidance of subsequent reintroduction [116]. In cases where amiodarone remains clinically necessary and no alternatives are available, cautious consideration can be given to its oral form with a reduced dosage (not exceeding 200 mg/day), while maintaining vigilant monitoring of liver function [116].

### 5.4. Thyroid

Amiodarone usage leads to changes in serum thyroid hormone levels, with typical alterations including decreased serum T3, increased serum T4 and reverse T3 levels, alongside normal or slightly elevated serum TSH [117]. Early elevations in serum TSH, often dose- and time-dependent, tend to normalize within a few months [118]. The impact of amiodarone on thyroid function stems from both inherent drug effects and its iodine content, although the latter alone does not account for the entire range of thyroid-related abnormalities observed in patients initiated on this medication [119,120].

#### 5.4.1. Amiodarone-Induced Hypothyroidism (AIH)

Amiodarone’s rich iodine content can lead to the Wolff–Chaikoff effect, inhibiting thyroid hormone synthesis and release, which is usually followed by an escape from this effect, resulting in normal T4 and T3 synthesis [121]. Persistent AIH can result from an enhanced susceptibility to iodine’s inhibitory effect, failure to escape the Wolff–Chaikoff effect, or both [122]. Hypothyroidism may resolve after stopping amiodarone, but persistent cases are often linked to underlying autoimmune thyroid disease, notably Hashimoto’s thyroiditis. The risk of amiodarone-induced hypothyroidism is higher in women, especially those with pre-existing thyroid antibodies [123]. Enhanced autoimmunity might explain this difference. Anti-thyroid peroxidase (Anti-TPO) antibodies are common, with a higher prevalence in women. The relationship between amiodarone’s iodine and autoimmune response is not conclusively established, and it is plausible that excess iodine contributes to thyroid injury alongside underlying autoimmune disease [117].

Hypothyroidism can arise within weeks to months (up to 39 months) after starting amiodarone, with serum TSH initially rising but later returning to baseline or lower levels [124]. Vague symptoms resembling spontaneous hypothyroidism, such as fatigue, cold intolerance, slow speech, dyspnea on exertion, and reduced exercise capacity, are common, making it challenging to distinguish them from cardiopulmonary issues [125]. Amiodarone’s noncompetitive β-blockade can lead to bradycardia, which may be worsened by hypothyroidism. QT/QTc interval prolongation occurs with both amiodarone and hypothyroidism, but the risk of severe arrhythmias is relatively low [126].

Due to its high efficacy, amiodarone is frequently the sole antiarrhythmic option available to patients, and it is commonly administered alongside levothyroxine (L-T4) replacement. L-T4 is preferred, especially for individuals with cardiac issues, due to its once-daily dosing and lack of serum thyroid hormone concentration spikes seen with liothyronine (L-T3) or desiccated thyroid treatment (a T4 and T3 mixture) [124]. Amiodarone users may need higher L-T4 doses to restore their serum TSH levels to normal, attributed to amiodarone’s impact on T4 to T3 conversion. Given the often-severe underlying cardiac conditions of these patients, maintaining serum TSH levels in the upper half of the normal range is advisable [119].

In myxedema coma, a medical emergency, prompt thyroid hormone replacement and supportive care are crucial, with respiratory support being pivotal. Rapidly increasing thyroid hormone levels carries some risk but is warranted due to the severe risks of myxedema coma [127]. There is no consensus on the best thyroid hormone replacement method, but IV administration is often preferred initially due to the unpredictability of intestinal absorption. Amiodarone-induced myxedema treatment poses a risk of secondary adrenal insufficiency, necessitating glucocorticoid therapy in stress dosage until adrenal issues are ruled out [128,129].

#### 5.4.2. Amiodarone-Induced Thyrotoxicosis (AIT)

Amiodarone-induced hyperthyroidism presents as a more intricate condition than AIH. Two primary forms of amiodarone-induced thyrotoxicosis (AIT) exist [130].

Type I AIT commonly arises in abnormal thyroid glands and results from excessive iodine-triggered hormone synthesis and release [131]. Disruption of the thyroid’s iodine handling due to dysregulated autoregulatory mechanisms is indicated by higher glandular iodine content in AIT compared to euthyroid amiodarone users, which normalizes as thyrotoxicosis resolves. This form is often linked to toxic nodular goiter or Graves’ disease in patients with pre-existing thyroid disorders [130,131].

In contrast, Type II AIT stems from thyroiditis causing the release of preformed thyroid hormones from damaged follicular cells and typically occurs in individuals without prior thyroid conditions [132]. Humoral thyroid autoimmunity has limited involvement in Type II AIT development among patients without pre-existing thyroid disorders. The prevalence of these AIT types is not well-defined, possibly influenced by ambient iodine intake [133]. Type II AIT endures for 1–3 months, depleting thyroid hormone stores, and responds more swiftly to glucocorticoid treatment [106]. A risk index for predicting amiodarone-induced thyrotoxicosis has been formulated for congenital heart disease patients, factoring in amiodarone initiation age, BMI, and the presence of cyanosis, but its relevance has not been established in other patient groups [134,135].

Clinical manifestations like unexplained weight loss, proximal myopathy, exacerbated arrhythmias or angina, and heat intolerance might raise suspicions of AIT. However, classical thyrotoxicosis symptoms could be absent due to amiodarone’s antiadrenergic impact and its hindrance on T4 to T3 conversion [132,133]. The presence or absence of a goiter, with or without thyroid region pain, can vary. Unless AIT coincides with Graves’ disease, goiter and ophthalmopathy are usually not observed [114]. Commonly, an abrupt resurgence of previously managed atrial and/or ventricular tachyarrhythmias serves as the predominant presentation. In more severe cases, electrical storms involving frequent VT or VF requiring cardioversion or defibrillation may arise [126]. AIT can also induce angina [6]. In patients with recurrent AF, managing thromboembolic events can be particularly complex. While thyrotoxicosis impacts the balance of coagulation and fibrinolysis, elevating thromboembolic risk, it also heightens warfarin sensitivity in those receiving it, irrespective of the thyroid disorder origin [136]. Hyperthyroid individuals demonstrate amplified depression of clotting factors (II, VII, IX, and X) and prolonged prothrombin times due to warfarin [137]. Amiodarone compounds this effect by hindering warfarin plasma clearance. This effect stems from the competitive inhibition of the hepatic CYP2C9 and vitamin K epoxide reductase subunit 1 gene (VKORC1) [138].

AIT is characterized by decreased serum TSH levels, along with elevated thyroxine, free thyroxine, and free thyroxine index levels. Some patients might also exhibit elevated serum levels of T3, free T3, and free T3 index [130]. Notably, T3 levels can remain within the normal range for up to 80% of patients [120].

Interruption of amiodarone treatment has been linked to worsened thyrotoxicosis, likely due to the loss of amiodarone-induced intracellular hypothyroidism [139]. Amiodarone’s lipophilic nature results in prolonged tissue storage, extending the duration of thyrotoxicosis even after discontinuation, which can persist for up to 8 months [117]. In cases where amiodarone has been effective in managing life-threatening arrhythmias, it is advisable to continue its use while treating hyperthyroidism. Switching to an alternative drug might be considered if feasible, but the long half-life of amiodarone delays any immediate benefits from discontinuation [117,139].

Radioactive iodine treatment is not a practical option for AIT in areas with sufficient iodine levels, as the radioiodine uptake is hindered by the high concentrations of intrathyroidal iodine. While its use has been described in Europe for Type I AIT with higher radioiodine uptake values, it remains unsuitable in iodine-sufficient regions like the United States [120].

Distinguishing between Type I and Type II AIT is crucial due to its therapeutic implications. Although serum thyroglobulin levels are often elevated in AIT, they may not be a reliable marker in goitrous patients with thyroid destruction [131]. ESR and C-reactive protein measurements have also proven unhelpful in discriminating the two AIT subtypes. The presence of thyrotropin receptor antibodies (TRAbs) indicates Graves’ [140]. Interleukin-6 (IL-6) is a promising marker, being normal or slightly elevated in Type I AIT and significantly raised in Type II AIT. However, elevated IL-6 levels can also occur in non-thyroidal conditions [119]. Color flow Doppler sonography of the thyroid is effective in distinguishing AIT types, with the absence of vascularity and glandular destruction being characteristic of Type II AIT [141]. Technetium-99m sestamibi (99mTc-STS) thyroid scintigraphy can differentiate AIT subtypes, although it relies on qualitative and subjective data. Recent use of quantitative thyroid-to-background ratios on a time-activity curve has improved interobserver reliability in assessing AIT types [141,142].

Type I AIT, caused by excessive hormone synthesis, is treated with thionamide drugs. Due to elevated intrathyroidal iodine levels, higher doses of thionamides are often required for effective therapy [139]. Thionamides (methimazole, carbimazole, propylthiouracil) inhibit thyroid peroxidase, reducing T3 and T4 synthesis [117]. Potassium perchlorate, used since the 1950s, inhibits the sodium/iodide symporter, blocking iodide transport into the thyroid and enhancing thionamide efficacy by depleting iodine stores. Perchlorate accelerates the attainment of euthyroidism compared to conventional thionamides [143]. However, it is no longer available in the United States due to toxicity concerns, including agranulocytosis, aplastic anemia, and renal dysfunction [144]. While short-term perchlorate use may heighten the risk of recurrent thyrotoxicosis, discontinuing it upon achieving euthyroidism is prudent [119]. Complications such as agranulocytosis and aplastic anemia are also rare with thionamide monotherapy, with a prevalence of 0.1–0.5% [145]. In some cases, a limited number of patients have received lithium carbonate to expedite the return to normal thyroid function. Lithium enhances intrathyroidal iodine levels, hinders the coupling of iodotyrosine residues for T4 and T3 formation, and restrains the release of T4 and T3 [146].

Type II destructive thyroiditis can not be effectively treated with thionamides, with or without potassium perchlorate. However, glucocorticoids are a successful treatment for Type II AIT due to their anti-inflammatory and membrane-stabilizing properties. Steroids also inhibit 5′-deiodinase activity [139]. Prednisone is initiated and gradually reduced over two to three months. During the taper, exacerbations may arise and require an increased steroid dosage [141]. Patients might experience transient hypothyroidism as thyrotoxicosis resolves, necessitating thyroid hormone replacement. When dealing with patients having mixed AIT forms or uncertain differentiation, a combination of thionamides and steroids (potentially with potassium perchlorate) is a promising therapeutic approach. Plasmapheresis might be considered for cases resistant to conventional treatment [119].

Beta-blockers alleviate thyrotoxicosis symptoms linked to heightened beta-adrenergic activity [141]. In the absence of reasons to avoid them, beta-blockers are recommended for initiation in most patients upon diagnosis. While certain beta-blockers slow down the action of 5′-monodeiodinase, which converts T4 to T3, this effect is gradual (taking 7 to 10 days) and has minimal impact on their therapeutic effectiveness [141].

Thyroid storm is a critical endocrine emergency with an incidence of 1–10%, more commonly affecting women and those with Graves’ disease. Mortality ranges from 10% to 30%, primarily due to multiorgan failure [147]. Supportive therapy addresses hyperthermia, heart failure, tachyarrhythmias, and more. Beta-blockers are a key treatment, alongside thionamides given at higher doses, and non-radioactive iodine administration (at least 30 min after thionamides, to avoid acting as a substrate for de novo thyroid hormone synthesis) to decrease hormone synthesis and release. Lithium can be an alternative to iodine [147,148]. Cholestyramine promotes thyroid hormone excretion by interfering with the enterohepatic circulation of these hormones [149]. Plasmapheresis or plasma exchange can rapidly reduce hormone levels and improve symptoms. Thyroxine-binding globulin, along with attached thyroid hormone, is removed from circulation, and albumin, typically used as a replacement, offers unoccupied binding sites for free thyroid hormone in circulation [150]. Although albumin binds thyroid hormone with less affinity compared to thyroxine-binding globulin, it has a higher capacity for low-affinity binding, leading to reduced concentrations of free thyroid hormone [151].

Thyroidectomy is typically reserved for severe, life-threatening thyrotoxicosis cases, including those induced by amiodarone or electrical storms. In cases where long-term amiodarone discontinuation is not feasible, surgical options are considered [141,152]. Surgical candidates include those who deteriorate despite medical treatment, experience severe side effects, or require rapid resolution due to cardiac or pulmonary issues [149]. Surgery (total or near-total thyroidectomy) quickly controls thyrotoxicosis and allows amiodarone therapy to continue. Full thyroidectomy is favored over partial, according to recent French data, though few AIT cases were included [153]. Surgical mortality in thyroid storm can approach 10%, while surgery appears relatively safe for AIT patients with cardiac disease. Some patients may use small beta-blocker doses before surgery, with caution due to potential synergistic effects with amiodarone [154].

### 5.5. Skin

*Phototoxic and photoallergic reactions.* Amiodarone-induced dermatological adverse effects are primarily phototoxic reactions, affecting 25–75% of long-term users. Photoallergy is less common but increases with prolonged therapy [6]. Amiodarone and its metabolite reduce the minimal erythema dose (MED) for both ultraviolet (UV) A and UVB radiation, likely due to free radical production that damages DNA and cell structures. Skin changes appear after at least 4 months of therapy, often on sun-exposed areas, with an erythematous or eczematous appearance, accompanied by pruritus [155]. Symptoms last up to 72 h and may persist even after amiodarone cessation due to its long elimination time [155]. Treatment involves drug withdrawal, restoring skin lipids, using topical steroids, non-steroidal anti-inflammatories, and sunscreen. Prevention includes limited sun exposure and using high sun protection factor (SPF), broad-spectrum sunscreens containing zinc oxide or titanium dioxide [155,156].

*Hyperpigmentation.* Amiodarone-induced skin hyperpigmentation affects 4–9% of patients, primarily those with skin type I. It results in blue-grey discolorations on the face, ears, and hands due to amiodarone deposits [157]. Histopathologically, lipofuscin aggregates and inflammatory cells are observed [158]. Hyperpigmentation occurs around 20 months into treatment, often with higher doses (400–800 mg/day) than in phototoxic reactions. Discontinuing the drug gradually reduces symptoms, achieving full remission after months to years [159]. Preventive measures include sun protection and possibly switching to another antiarrhythmic drug. Treatments like vitamin B6, depigmentation agents, and lasers have shown varied success. UVB-rays therapy might improve tolerance to sunlight [158,159].

*Pseudoporphyria.* Amiodarone is implicated in causing pseudoporphyria, a condition resembling porphyria cutanea tarda without corresponding porphyrin metabolism changes [160]. Pseudoporphyria displays symptoms like tense bullae, erosions, scars, hyperpigmentation, and heightened skin fragility, particularly in sun-exposed areas [156].

*Linear IgA Bullous Dermatosis.* An extremely uncommon adverse response to amiodarone therapy is linear immunoglobulin A (IgA) bullous dermatosis, and it is believed that amiodarone might worsen the progression of this condition [161]. Distinguishing features include well-defined blisters found on either unaffected skin or on reddened and swollen regions. The affected areas do not necessarily align with sun-exposed skin portions [156,161].

*Other cutaneous side effects.* Less frequent skin-related issues stemming from amiodarone use include urticaria, itching, erythema nodosum, purplish discoloration, and the severe form of erythema multiforme known as toxic epidermal necrolysis [162,163]. A sudden complication arising from the IV administration of amiodarone can result in skin and soft tissue necrosis due to leakage into veins. The risk is linked to the solution’s low pH (3.5–4.5) and the presence of solvents like benzyl alcohol and polysorbates [164]. Some reports suggest potential carcinogenic effects of amiodarone, with a few cases linking chronic use of the drug to basal cell carcinoma [165].

### 5.6. Eyes

Chronic amiodarone therapy often leads to keratopathy due to micro-deposits in the cornea, which can develop within the first few months of treatment [166]. Keratopathy is classified using a grading system proposed by Orlando et al. [167]. Although usually asymptomatic, some patients may experience ocular irritation, photophobia or halos [168]. This deposition has been suggested as a useful marker for monitoring amiodarone therapy and toxicity through corneal densitometry [169]. These deposits typically clear within 3–20 months after stopping the drug. Since they rarely cause significant symptoms, discontinuing amiodarone is generally not required [170].

Amiodarone-induced optic neuropathy is a rare but serious condition characterized by a gradual onset of visual loss, bilateral involvement, and protracted resolution after drug discontinuation [171]. Diagnosis relies on parameters such as the onset of visual loss, severity, resolution time, and ocular involvement [172]. It shares features with non-arteritic anterior ischemic optic neuropathy, differing in sex distribution, laterality, optic disk appearance, and duration of disk edema [171]. The condition requires monitoring by an ophthalmologist due to the potential for severe vision loss. However, a direct causal link between amiodarone and optic neuropathy remains speculative [173].

### 5.7. Other Side Effects

*Nervous system*. Amiodarone can cause several side effects on the nervous system, including peripheral neuropathy, tremors, and ataxia [174]. Insomnia, cognitive impairment, and even nystagmus are also reported, although rare [175]. The occurrence and severity of these side effects can vary depending on individual patient sensitivity, dosage, and duration of treatment. The importance of recognizing neurological side effects cannot be overstated, as they are uncommon and frequently underreported or overlooked [176].

*Gastrointestinal system.* Amiodarone may lead to gastrointestinal side effects such as nausea, vomiting, decreased appetite, and constipation, which are frequently observed in patients on long-term therapy. These symptoms often improve when the dosage is reduced [177].

*Muscular system*. Amiodarone may cause muscular side effects, such as myopathy and muscle weakness, particularly with prolonged use [178]. The most common clinical manifestation is primarily proximal myopathy, while in extremely rare instances, acute necrotizing myositis may develop [179].

*Genitourinary system.* In rare instances, amiodarone may lead to epididymitis, orchitis, and erectile dysfunction [180]. Amiodarone-induced epididymitis typically manifests as chronic pain in the epididymis without signs of fever or elevated WBC count. The recommended treatment involves either lowering the dosage of amiodarone or discontinuing the medication altogether [181].

**Figure 1:**
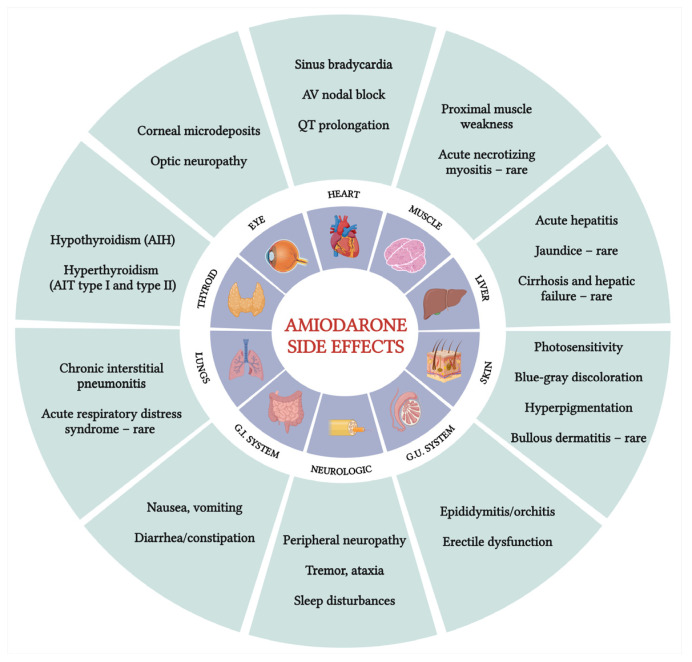
Amiodarone side effects. AV, atrioventricular; AIH, amiodarone-induced hypothyroidism; AIT, amiodarone-induced thyrotoxicosis; G.I., gastrointestinal; G.U., genitourinary. (Created with BioRender.com; accessed on 9 September 2024 ).

Amiodarone side effects are summarized in Figure 1.

## 6. Monitoring

**Figure 2:**
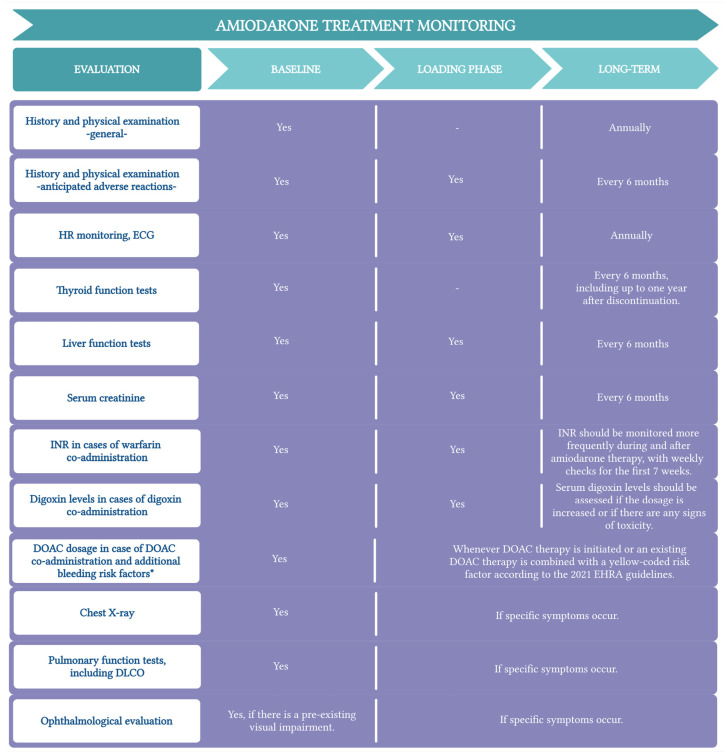
Amiodarone treatment monitoring. Adapted from The North American Society of Pacing and Electrophysiology [183] and NHS Derbyshire Joint Area Prescribing Committee recommendations [184]. * Yellow-coded risk factors according to the 2021 EHRA guidelines; HR, heart rate; ECG, electrocardiogram; DLCO, diffusing capacity of the lungs for carbon monoxide; EHRA, European Heart Rhythm Association. (Created with BioRender.com; accessed on 9 September 2024).

Individuals undergoing amiodarone therapy need periodic monitoring to evaluate the ongoing necessity of the drug, its effectiveness, appropriate dosage, adverse reactions, and possible interactions with other medications (Figure 2).

Despite the known side effects and frequent use of amiodarone, few guidelines exist for monitoring patients receiving outpatient treatment with this drug [16,182,183,184]. Existing guidelines generally agree on four categories of tests: initial baseline tests at the start of treatment, loading phase tests, routine tests at regular intervals (either annually or every six months), and specific tests triggered by symptoms suggesting side effects [106].

## 7. Conclusions

Amiodarone is a potent antiarrhythmic drug commonly used for treating both ventricular and atrial arrhythmia in ordinary practice. Taking into consideration the widespread use of this medication, it is necessary for practitioners to be well-informed about its indications, contraindications, dosing recommendations, side effects, and potential drug interactions. Despite its effectiveness, amiodarone is associated with a wide range of potential adverse effects, from well-studied issues like thyroid dysfunction and pulmonary toxicity to less commonly recognized effects, such as neuromuscular toxicity. This underscores the importance of proper and constant evaluation of the patients through their treatment. Regular patient evaluation not only ensures optimal dosing and reduces the risk of drug interactions but is also critical for the early identification and management of any adverse effects.

## Figures and Tables

**Table 1:** Amiodarone indications as a first- or second-line therapy reported by the cited guidelines.

Indication	First-Line AADs	Second-Line AADs	Reference
Cardiac arrest VF Hemodynamically unstable VT	IV amiodarone IV lidocaine	N/A	[19]
Stable VT	IV verapamil—fascicular VT IV beta-blockers—outflow tract VT IV procainamide	IV amiodarone IV flecainide IV sotalol	[20]
AF—acute cardioversion	IV flecainide IV propafenone	IV amiodarone IV vernakalant	[21]
AF—acute rate control	IV beta-blockers (metoprolol tartrate, esmolol, landiolol) IV verapamil IV diltiazem	IV amiodarone	[21]
Other supraventricular tachyarrhythmias—acute rate control	IV adenosine	IV amiodarone	[21]
Prevention of recurrent ICD shocks and VT	PO beta-blockers PO amiodarone	AAD according to underlying disease and cardiac function	[20]
AF—prevention of recurrent AF	PO amiodarone PO dronedarone PO flecainide PO propafenone	PO sotalol	[21]
AF—rate control	PO beta-blockers (metoprolol tartrate, metoprolol succinate, bisoprolol, atenolol, nebivolol, carvedilol)	PO verapamil PO diltiazem PO digoxin. PO amiodarone only as a last line.	[21]

AADs, antiarrhythmic drugs; VF, ventricular fibrillation; VT, ventricular tachycardia; AF, atrial fibrillation; ICD, implantable cardioverter-defibrillator; IV, intravenous; PO, per os; N/A, not applicable.

## Data Availability

Not applicable.
